# Infrared thermography in the detection of brown adipose tissue in humans

**DOI:** 10.14814/phy2.12167

**Published:** 2014-11-20

**Authors:** Christina Jang, Sandya Jalapu, Moe Thuzar, Phillip W Law, Susanne Jeavons, Johanna L. Barclay, Ken K.Y. Ho

**Affiliations:** 1 School of Medicine, University of Queensland, Brisbane, Qld, Australia; 2 Department of Endocrinology and Diabetes, Princess Alexandra Hospital, Brisbane, Qld, Australia; 3 Department of Medical Imaging, Princess Alexandra Hospital, Brisbane, Qld, Australia

**Keywords:** Brown adipose tissue, human, infrared thermography, thermogenesis

## Abstract

PET‐CT using ^18^F‐FDG is employed for detecting brown adipose tissue (BAT) in humans. Alternative methods are needed because of the radiation and cost of PET‐CT imaging. The aim was to evaluate the accuracy of infrared thermography (IRT) in detecting human BAT benchmarked to PET‐CT imaging. Seventeen individuals underwent a total of 29 PET‐CT scans, 12 of whom were studied twice, after 2 h of cold stimulation at 19°C, in parallel with measurement of skin temperatures overlying the supraclavicular (SCV) fossa and the lateral upper chest (control), before and after cold stimulation. Of the 29 scans, 20 were BAT positive after cold stimulation. The mean left SCV temperature tended to be higher in the BAT‐positive group before and during cooling. It was significantly higher (*P *=**0.04) than the temperature of the control area, which fell significantly during cooling in the BAT‐positive (−1.2 ± 0.3°C, *P *=**0.002) but not in the negative (−0.2 ± 0.4°C) group. The temperature difference (Δtemp) between left SCV and chest increased during cooling in the BAT‐positive (1.2 ± 0.2 to 2.0 ± 0.3°C, *P *<**0.002) but not in the negative group (0.6 ± 0.1 to 0.7 ± 0.1°C). A Δtemp of 0.9°C conferred a positive predictive value of 85% for SCV BAT, superior to that of SCV temperature. The findings were similar on the right. In conclusion, the Δtemp is significantly and consistently greater in BAT‐positive subjects. The Δtemp quantified by IRT after 2‐h cooling shows promise as a noninvasive convenient technique for studying SCV BAT function.

## Abstract (precis)

Using infrared thermography (IRT), we observed the skin temperature of the supraclavicular fossa (SCV) where brown adipose tissue (BAT) is present, to be higher than that of a control BAT‐negative chest area. This temperature difference was greater in subjects with BAT and conferred a positive predictive value of 85% for BAT benchmarked to PET/CT imaging. IRT shows promise as a tool for detecting BAT function in humans.

## Introduction

The recent years have seen a dramatic resurgence of interest in brown adipose tissue (BAT) in humans. Following the discovery of FDG‐avid depots in the supraclavicular and neck regions of humans undergoing Positron Emission Tomography (PET)‐CT scanning, investigations moved rapidly to histological confirmation of these depots as BAT (Zingaretti et al. 2009; Lee et al. 2011b) and then to define its contribution to energy expenditure. Indeed, efforts have now turned to exploring BAT as a novel therapeutic target in human metabolic disease.

Currently, PET‐CT is the gold standard method for the detection of BAT. However, PET‐CT has a number of limitations, including cost, radiation exposure, and duration of the procedure (Nedergaard and Cannon 2010; Lee et al. 2013). Diagnostic PET‐CT scans expose substantial radiation to the subject (Huang et al. 2009). While radiation exposure can be minimized in scans performed for research purposes, it will continue to limit the use of PET‐CT particularly for healthy volunteers. Furthermore, the reproducibility of PET‐CT imaging is poor (Lee et al. 2010) because of the susceptibility to changes and differences in scanning temperature and seasonal variations in temperature (Cohade et al. 2003; Au‐Yong et al. 2009; Zukotynski et al. 2009; Ouellet et al. 2011). Therefore, there is a need to develop other methods to investigate BAT physiology.

Because of the thermogenic properties of BAT, thermal imaging is a potential method for detecting BAT and studying its function. Using infrared thermography (IRT), we have previously reported a significantly higher skin temperature in the region overlying the supraclavicular (SCV) fossa than the mediastinal (MED) region in 87 individuals (Lee et al. 2011a). In one subject, this temperature difference became more pronounced following a meal ingestion and cold exposure, both known stimulants of BAT activity. Temperature increases within the supraclavicular region in response to cold exposure have also been confirmed with thermography in a recent study (Symonds et al. 2012). However, studies directly comparing and validating the results of IRT with PET‐CT imaging have not been published. The aim of this study was to assess the accuracy of IRT to predict the presence of BAT as benchmarked against PET‐CT imaging specifically to establish reproducibility, sensitivity, and specificity.

## Subjects and Methods

### Subjects

Seventeen healthy subjects (12 men and five women) were studied after obtaining written informed consents. The mean age (±SD) was 36 ± 8 years and BMI was 25.4 ± 5.9 kg/m^2^. Subjects were recruited to undergo PET‐CT scanning in a study to optimize conditions for the detection of BAT. In all subjects, PET‐CT scans were undertaken after a 6‐h fast and none had fasting hyperglycemia. The study was approved by the Human Research and Ethics Committee of Metro South Health Service.

### Methods

All subjects underwent a 2‐h period of cooling at 19°C in an air‐conditioned room prior to intravenous administration of ^18^F‐FDG. Of the 17 subjects, 12 subjects were studied twice 2–3 weeks apart. This was done to determine the reproducibility of BAT detection under the 2‐h precooling conditions, collectively providing a total of 29 scans for analysis. We previously reported that the reproducibility of BAT detection under ambient conditions for routine diagnostic PET scanning (for malignancy staging) is low, being less than 15% (Lee et al. 2010). We treated each measurement as separate cases because the brown fat activity may vary within the same individual between different occasions of scanning. It was therefore important to determine whether the IRT measurements correspond to BAT activity on PET scans on every occasion.

#### PET‐CT imaging

All subjects were seated and rested comfortably in a quiet room during the procedure. PET imaging was undertaken at 1 h after intravenous injection of 75 MBq (2 mCi) of ^18^F‐FDG on a *Biograph mCT 128* (Siemens Healthcare, Erlangen, Germany) equipped with time‐of‐flight electronics, in three‐dimensional list mode for 30 min in one bed position covering the skull base to the aortic arch. Noncontrast low‐dose CT (80 mAs) was subsequently performed for attenuation correction and localization of FDG‐avid sites. PET and CT image datasets were reconstructed in axial, coronal, and sagittal planes with a slice thickness of 4 mm. Images were interpreted by a dual‐qualified radiologist/nuclear physician using *syngo.via* software (Siemens Healthcare). BAT volume (in cm^3^) was quantified for right SCV and left SCV areas by autocontouring of FDG uptake above a set threshold (SUV_max_ 1.5) that show fat attenuation on CT. PET‐CT images for BAT were classified as “BAT Positive” or “BAT Negative” accordingly.

#### Infrared thermography

Subjects were seated in the upright position in an arm chair with head positioned in a neural position and the subject looking straight ahead. The upper torso from the chest area to neck region was exposed. A thermal imaging camera (FLIR B425, 3.1Mpixel, FLIR Systems Australia Pty Ltd, Melbourne, Vic., Australia) was used to acquire images of the anterior neck and upper chest region. The camera was positioned at the level of the neck 1 m from the subject's face. IRT was performed on all subjects at baseline ambient room temperature and then at 60 and 120 min during cooling at 19°C prior to PET‐CT scanning.

**Figure 1.**
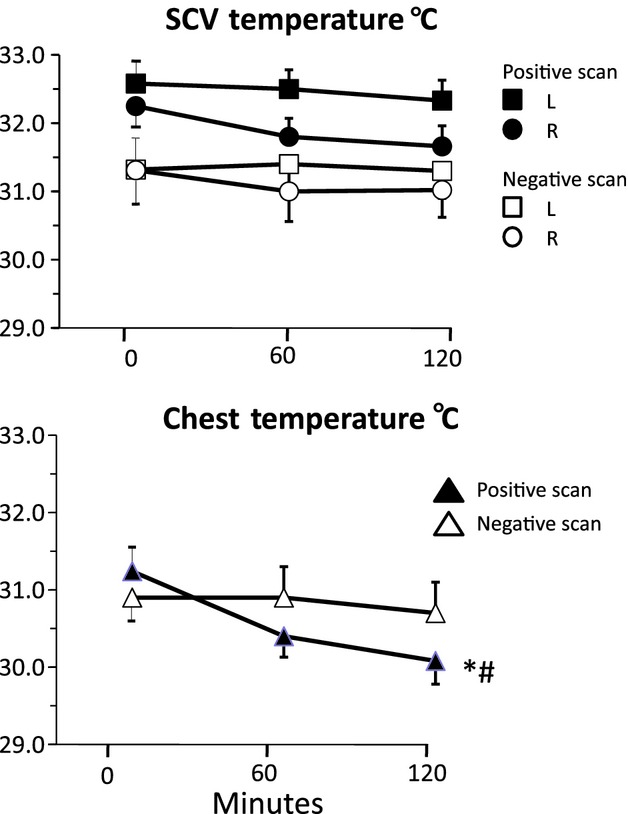
Mean (±SE) temperatures over the left (L) and right (R) supraclavicular areas (upper panel) and the anterior chest (lower panel) over 2 h of cooling at 19°C in an air‐conditioned room in subjects with positive and negative PET scans. *P <**0.05 for fall in temperature with time; #*P *<**0.05 for difference between PET‐positive and ‐negative groups.

Using FLIR Research IR Professional Analyzing Software (Version 1.2, Wilsonville, OR), skin temperatures overlying the SCV fossa bilaterally and an area in the upper chest just lateral to the sternum approximating the second intercostal space (control) were determined for each image. Analyses were performed for both right and left SCV regions (Fig. 1).

For thermal analysis, the camera was set to fusion mode which displays a fusion of digital and thermal images. The emissivity was set to 0.98 for skin as detailed by the FLIR operations manual.

Temperature measurement of the SCV region was performed as follows. A circle, 2 cm margins, centered immediately above the midclavicle was selected for temperature analysis. In instances where there is a temperature gradient peak in the SCV fossa, there is a color gradation of increasing temperature from the periphery to the middle of the area. A target region of measurement was set around the first temperature contour gradient from the periphery and the highest temperature within this target area was recorded for later analysis. In images where no temperature gradient is apparent, an area above the midclavicle corresponding to the SCV fossa was selected for temperature measurement. This procedure was repeated for the chest control area. There was no difference in skin temperatures of the chest control areas on the left and the right. Analyses were undertaken using only the chest control readings on the left. iButtons are available as a means of recording skin temperature directly. iButtons were not used in this study because preliminary evaluation revealed excellent concordance between the camera and iButtons readings.

#### Statistical analysis

Statistical analysis was performed using SPSS version 21 (SPSS Inc., Chicago, IL, USA). Data are reported as mean ± SEM. The unpaired *t*‐test was used to compare data between the BAT‐positive and BAT‐negative groups. Two‐way repeated measures ANOVA was employed to determine whether temperatures changed significantly during cooling and whether this was influenced by PET status. Pearson correlation analysis was employed to test the relationships between different variables. Differences in temperatures during cooling between PET‐positive and PET‐negative groups were determined by two‐way ANOVA. The sensitivity and specificity of IRT were determined by ROC analysis. A *P* value <0.05 was considered significant.

## Results

Of the 29 scans performed after precooling, 20 were positive for BAT and nine were negative (Table 1). Of the 12 subjects who were studied twice, 10 had concordant scans (seven repeat positive, three repeat negative scans) while two subjects had discordant scans. 15 participants had repeat thermography images taken twice within 1 min on at least one occasion. The mean difference in recorded temperature between two images for 15 subjects was 0.06 ± 0.01°C with a CV of 0.009%.

**Table 1.** Clinical characteristics of subjects categorized to BAT status as defined by PET‐CT imaging. Data are expressed as Mean ± SEM

	BAT Positive	BAT Negative	*P* value
Number of scans	20	9	
Gender	9M, 2F	3M, 3F	NS
Age (years)	31.7 ± 1.7	40.6 ± 5.0	0.06
BMI (kg/m^2^)	24.5 ± 1.1	27.2 ± 3.9	NS
SCV BAT vol (cm^3^)			
Left	47.4 ± 17.3	0	
Right	43.3 ± 17.1	0	

M, male; F, female; BMI, body mass index.

### SCV temperature

At baseline before cooling, mean left SCV temperature in the PET positive tended to be higher than that in the PET‐negative group (32.8 ± 0.3 vs. 31.5 ± 0.5, *P *=**0.055) (Fig. 1). A similar trend was present on the right (32.4 ± 0.3 vs. 31.4 ± 0.6, *P *=**0.06).

After 2‐h cooling, the mean SCV temperature remained higher in PET‐positive than PET‐negative group on both sides (left 32.3 ± 0.3 vs. 31.3 ± 0.5, *P *=**0.08 and right 31.6 ± 0.3 vs. 31.0 ± 0.5, *P *=**0.1), but the differences did not reach statistical significance nor did the temperature change significantly over the 2‐h cooling period (two‐way ANOVA) (Fig. 1).

Comparing left and right SCV temperatures, the mean skin temperature after 2‐h cooling overlying the left SCV was significantly higher than that on the right for both PET‐positive (32.3 ± 0.3 vs. 31.6 ± 0.3°C, *P *<**0.001) and PET‐negative groups (31.3 ± 0.5 vs. 31.0 ± 0.5°C, *P *<**0.01).

The mean BAT volume in the left SCV fossa was not significantly different to that of the right on the PET scan. SCV temperatures did not correlate significantly with SCV BAT volume.

### Control chest temperature

At baseline before cooling, there was no difference in the mean chest temperatures between PET‐positive and PET‐negative groups (31.2 ± 0.4 vs. 30.9 ± 0.4) (Fig. 1).

After cooling, the mean chest temperature fell significantly by 1.2 ± 0.3°C (p = 0.002) in the PET‐positive but not in the PET‐negative (0.2 ± 0.4°C) group (Fig. 1) and the difference was significant between the groups (*P *=**0.04). As cooling induced a greater fall in chest than in SCV temperatures, this resulted in a rise in the magnitude of temperature difference (∆temp) between the two sites.

### Temperature difference between SCV and control chest areas (∆temp)

**Figure 2.**
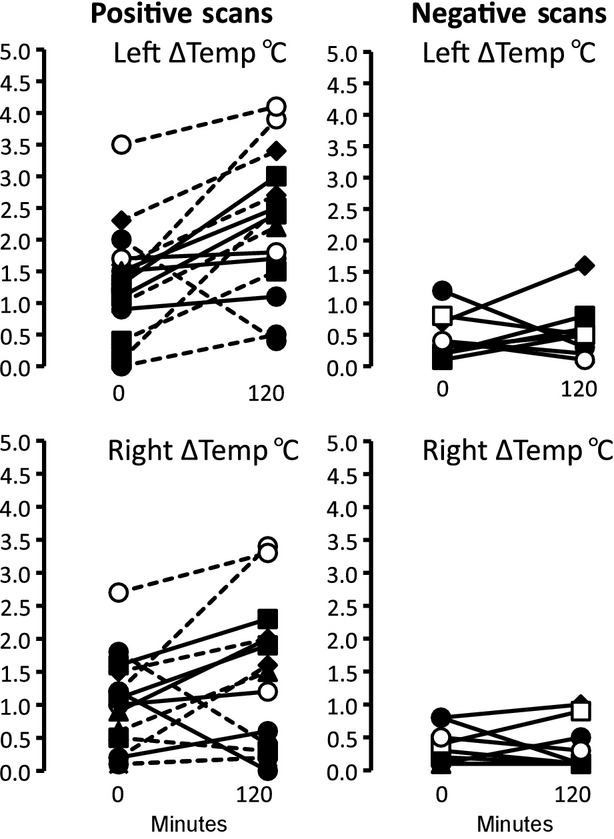
Upper panel shows Individual paired plots in PET‐positive (left panels) and PET‐negative (right panels) subjects studied before and after 2 h of cooling. The temperature difference (∆temp) between supraclavicular (left and right) and chest areas was measured. Each subject is denoted by a different symbol. The same symbols are used for subjects studied on a second occasion.

At baseline before cooling, in the BAT‐positive group, the mean SCV temperatures on both sides were significantly higher than the control chest temperature (Fig. 1). Similarly, in the BAT‐negative group, the mean SCV temperatures on both sides were also significantly higher than mean chest temperature. ∆temp was greater in the BAT positive than in the negative group on the left (1.2 ± 0.2 vs. 0.6 ± 0.1°C, *P *=**0.04) and right sides (1.0 ± 0.2 vs. 0.5 ± 0.01°C, *P *=**0.04) (Fig. 2). The ∆temp was significantly greater on the left than on the right side (*P *=**0.004) in the BAT‐positive group.

After cooling, ∆temp between SCV and chest areas remained significantly greater in the BAT positive than the negative group on the left (2.1 ± 0.3 vs. 0.7 ± 0.1°C, *P *=**0.002) and the right side (1.4 ± 0.3 vs. 0.4 ± 0.1°C, *P *=**0.02). Moreover, for the 20 positive scans, the SCV BAT volume was significantly correlated with ∆temp which was stronger on the left side (*r* = 0.57, *P *=**0.004) than on the right (*r* = 0.49, *P *=**0.02).

∆temp on the left rose significantly (1.2 ± 0.2 to 2.1 ± 0.3 °C; *P *= 0.04) in the BAT‐positive group but not in the BAT‐negative (0.6 ± 0.1 to 0.7 ± 0.1°C, *P* = 0.6) group (Fig. 2) with cooling. The change in ∆temp on the right was similar, increasing in the BAT‐positive (1.0 ± 0.2 to 1.4 ± 0.3°C, *P *= 0.09) but not in the BAT‐negative (0.5 ± 0.1 to 0.4 ± 0.1°C) group. The ∆temp change was greater on the left than right side throughout cold stimulation in the BAT‐positive group (*P* = 0.01). Figure 2 shows paired plots of ∆ temp on the right and left sides for each of the assessments in PET‐positive and PET‐negative scans.

**Figure 3.**
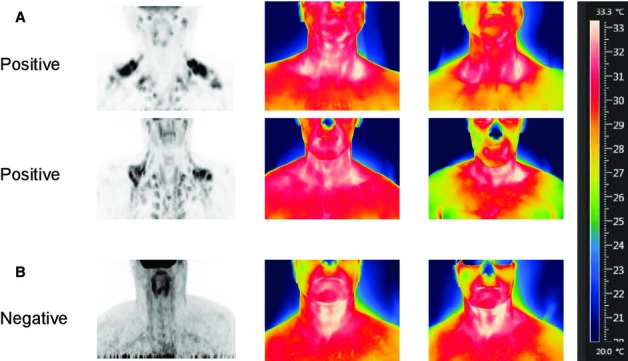
Representative PET‐CT and thermography images before and after 120‐min cooling for (A) two BAT‐positive subjects and (B) one BAT‐negative subject. In A, the thermograms for both PET‐positive subjects indicate that the skin temperatures in the SCVs are higher than the surrounding areas and with cooling, there is a fall in chest temperature as denoted by the color change. (B) Shows minimal difference in temperature between SCV and control chest areas. With cooling, there is no fall in chest temperature.

Representative PET/CT scans and corresponding thermograms before and after cooling from two PET‐positive and a PET‐negative subject are shown in Figure 3. For the two positive scans, SCV temperatures are higher than the adjacent lateral areas before and after cooling. Note the fall in chest temperature after cooling. In the PET‐negative subject, the temperature in the SCV fossa is similar to the adjacent lateral area. There is no change in chest temperature after cooling.

#### ROC analysis

Before cooling, ROC analysis revealed that an optimal discriminant ∆temp of >0.9 conferred a sensitivity of 77% and specificity of 78% on the left for a positive PET scan (Table 2). After 2 h of cooling, sensitivity increased to 87% with specificity unchanged. On the right, before cooling a Δtemp of ≥ 0.9°C conferred a sensitivity and specificity figures of 59% and 89% and after cooling, 68% and 78%, respectively. The sensitivities and specificities of SCV temperatures on the left and right were inferior to those of the corresponding ∆temps. The corresponding positive and negative predictive values displayed in Table 2 show that the ∆temp of 0.9 on the left side at 2 h of cooling confers a positive predictive value of 85% and negative predictive value of 77% for a positive PET scan. The sensitivity, specificity, and predictive values for SCV temperatures were inferior to corresponding ∆temps regardless of the side of analysis.

**Table 2.** Sensitivity and predictive value of infrared thermography for a positive PET/CT scan

	Time (min)	Discriminant (°C)	Sensitivity (%)	Specificity (%)	PPV (%)	NPV (%)
Left SCV	0	32.6	76	78	72	89
	120	32.6	64	67	61	67
Right SCV	0	31.6	76	67	77	56
	120	31.6	65	67	61	67
Left Δ temp	0	0.9	77	78	72	77
	120	0.9	87	78	85	77
Right Δ temp	0	0.9	59	89	60	88
	120	0.9	68	78	65	67

PPV, positive predictive value; NPV, negative predictive value.

Skin temperatures were measured over the supraclavicular (SCV) areas and the anterior chest at 0 and 120 min of cooling in an air‐conditioned room. ∆temp refers to the skin temperature difference between SCV and the anterior chest. The discriminant temperatures that conferred the optimal sensitivity and specificity were determined by ROC analysis.

## Discussion

IRT is an established and accepted technique for studying BAT function in small animals (Mccafferty 2007; Marks et al. 2009; Gilbert et al. 2012; Warner et al. 2013). Studies employing IRT to assess BAT activity in humans are beginning to emerge (Lee et al. 2011a; Symonds et al. 2012). However, the predictive value for detecting BAT by IRT in humans has not been rigorously assessed. This study investigated the usefulness of IRT as a tool for detecting BAT in humans by validating thermal imaging to that of positive PET‐CT imaging. During controlled cold stimulation in an air‐conditioned room, the mean skin temperature over the SCV fossa tended to be higher in the BAT‐positive group. Mean SCV temperatures were consistently higher than that over the chest in both BAT‐positive and BAT‐negative groups. The temperature difference between SCV and chest areas was significantly greater in the BAT‐positive group, correlated significantly with BAT volume and was consistently greater on the left. This temperature difference increased during cooling, an effect resulting from a fall in chest temperature rather than a rise in SCV temperature. The ∆ temp after cooling correlated significantly with BAT volume. The temperature difference was a better predictor of PET status than SCV temperature.

The observation that BAT‐positive subjects exhibited a fall in chest temperature compared to BAT‐negative counterparts is intriguing. It is conceivable that skin temperature over the chest provides afferent sensory input for the central activation of the SNS required to stimulate BAT function. Thus, a lower chest cutaneous temperature triggers a greater activation of BAT to maintain SCV temperature at a level which might have been lower in the absence of BAT activity. It is possible that the amount of body fat influenced the magnitude of the fall in chest temperature during cooling. Diminished thermal insulation could trigger the SNS, simultaneously stimulating BAT activity and causing peripheral skin vasoconstriction. As we did not measure body composition, we are unable to determine whether the proportion of body fat was lower in PET‐positive subjects.. Several studies have reported that body mass index is a negative predictor of BAT activity (Lee et al. 2013). In this study, mean body mass index tended to be lower among the PET‐positive subjects. Regardless of the mechanism(s), we have observed, in BAT‐positive subjects, higher SCV temperature, a greater skin temperature difference between BAT‐positive and ‐negative sites that is significantly correlated with the mass of BAT. These findings provide strong evidence that BAT in adult human is thermogenic and detectable by IRT.

We undertook a systematic ROC analysis of the impact of cooling on the value of SCV temperature and the ∆temp in predicting BAT as identified by PET/CT. It is well established that cold stimulation enhances the detection and activity of BAT as assessed by PET‐CT imaging in humans. The rate of BAT detectability increases from 5–10% at ambient temperature to over 90% after cold stimulation (Lee et al. 2011b), a change equating to an increase in BAT activity of 10‐ to 15‐fold (Virtanen et al. 2009). SCV temperature was far inferior to ∆ temperatures and cooling had little effect because SCV temperatures did not change. In contrast, cooling enhanced the predictive value of ∆temp on both left and right sides. Like PET‐CT, the diagnostic power of IRT is enhanced by cooling.

Another interesting observation was the consistently higher SCV temperature on the left than on the right, regardless of BAT status, and a greater temperature difference on the left between SCV and chest areas in BAT‐positive subjects. We observed no differences in SCV BAT mass between the sides nor asymmetry in the FDG uptake in those with positive scans suggesting that the lateralization is unlikely to be BAT related. The cooling technique is also unlikely to have had much bearing; subjects sat in an air‐conditioned room where cooling was spread evenly. Moreover, this temperature difference is evident before cooling and maintained throughout. We speculate that higher SCV skin temperature on the left arises from the unique vascular anatomy from a capacious brachiocephalic vein on the left.

IRT has several advantages. The procedure is convenient as it can be undertaken within minutes, unlike PET‐CT which requires a postinjection and scanning time of at least 2 h. Importantly, there is no radiation exposure with IRT, whereas for PET‐CT using a standard FDG dose, the radiation from a single study for oncological indications far exceeds radiation safety limits for investigative studies in normal subjects. Another useful application for IRT is in the monitoring of changes in BAT activity over minutes or hours that cannot be done with PET‐CT, which is restricted to a single time domain of capture over 1 h. In that regard, IRT offers considerable advantages as a safer and more flexible tool for studying BAT function and activity.

IRT conferred a probability of greater than 80% in predicting BAT. While promising, we caution against using IRT independent of PET‐CT imaging until more evaluations are published. At this stage, we propose that it serves as a screening tool for selecting subjects for regulatory studies of BAT function in whom the presence of significant BAT activity is initially confirmed by PET‐CT imaging. A baseline of PET positivity brings specificity to the interpretation of changes in temperature difference between the neck and chest as changes in BAT activity.

One of the limitations of IRT is that it is confined to studying BAT that is located superficially, particularly the SCV fossa. It is unlikely that thermogenicity of BAT located in deeper areas can be detected by IRT. Therefore, the study of BAT activity in humans by IRT is restricted to superficial BAT depots. Our study population is small and consisted of a heterogeneous group of individuals of varying age and BMI undergoing simultaneous PET‐CT scanning after cold stimulation by air conditioning. Future studies comprising larger numbers are required to provide more information on the value of IRT in the evaluation of BAT in humans.

This is the first study investigating the utility of IRT for detecting BAT referenced to PET‐CT. Our evaluation provides strong evidence that IRT is a promising noninvasive technique for the detection of BAT in the SCV region. The discriminant measure is the difference in skin temperatures between the SCV and the lateral chest areas, and not the temperature overlying the SCV fossa after cold stimulation. The predictive value of the temperature difference is better on the left than on the right. IRT is a useful technology that may complement PET‐CT imaging in the study of BAT in humans.

## Acknowledgments

We thank the staff of the Department of Medical Imaging at the Princess Alexandra Hospital, who performed the PET‐CT scans. We thank Dr. Paul Lee for providing input during the conduct of this study. Dr. Jalapu and Thuzar were supported by fellowships from the Princess Alexandra Research Support Scheme. This study was supported by the NHMRC of Australia.

## Conflict of interest

None declared.
